# Characteristics of the home environment in children with developmental delays: insights from a cross-sectional study in Türkiye

**DOI:** 10.55730/1300-0144.5956

**Published:** 2025-02-04

**Authors:** Emel ÖMERCİOĞLU, Ebru CİHAN ÇAM, Ömer Nazım GÜLÇEK, Elif N. ÖZMERT

**Affiliations:** 1 Division of Developmental Pediatrics, Department of Pediatrics, Faculty of Medicine, Hacettepe University, Ankara, Turkiye; 2 Department of Pediatrics, Dışkapı Yıldırım Beyazıt Education and Research Hospital, Ankara, Turkiye

**Keywords:** Developmental delay, children, home environment

## Abstract

**Background/aim:**

A stimulating home environment in early childhood plays a crucial role in fostering child development. This study aimed to evaluate the home environment of children with developmental delays and who had not received special education support in any developmental domain.

**Materials and methods:**

The study comprised of 50 preschool children with developmental delay monitored at Hacettepe University Division of Developmental Pediatrics and 70 typically developing preschool children from Ankara 29 Mayıs State Hospital, General Pediatrics Department. Physicians and experienced child development professionals employed the Ages and Stages Questionnaire (ASQ) to perform developmental assessments on each child. The characteristics of the home environment for all participants were evaluated using the Home Environment Questionnaire (HEQ), and their sociodemographic details were recorded.

**Results:**

The home stimulation scores for children with developmental delay were significantly lower compared to those for their typically developing peers (p < 0.001). Physical violence against children was reported by 34% of parents of children with developmental delays (p < 0.001). The presence of developmental delay (p < 0.001, CI 12.629–7.927), the family socioeconomic status (p = 0.002, CI 3.813–1.486), and the number of family members (p = 0.001, Cl 2.595–0.708) were significantly associated with home stimulation scores.

**Conclusion:**

Initial assessment of children with developmental delays suggest that the stimuli in their home environment are of lower quality relative to those of typically developing peers, and these children are subjected to markedly higher levels of physical violence. These results underscore the need for early childhood caretakers, public health practitioners, and other professionals to design and implement targeted interventions, particularly prioritizing low-income families living in disadvantaged environments.

## 1. Introduction

The World Health Organization (WHO) highlights the crucial role of the nurturing care framework in ensuring optimal development during early childhood. This framework includes the elements of health, nutrition, security and safety, responsive caregiving, and early learning opportunities [1]. In particular, early learning opportunities are provided through parent and family interactions, supported by an environment conducive to these interactions. The primary environment for children is their family and home setting, and the WHO stresses that the first three years of life are critically influenced by the home environment [2]. The home environment is characterized as a secure and well-organized physical setting that includes opportunities to play and explore, as well as learning materials, toys, and books [3].

The existing literature clearly establishes the link between child development and affordances [4]. Poor home environment stimulation raises the likelihood of cognitive, language, gross and fine motor, and social-emotional development delays in healthy infants and preschoolers, based on a variety of research [5–7]. The severity of the child’s physical or developmental disability, care priorities, and expectations from the child are a few of the factors that may prevent families with children who have developmental delays or disabilities from providing their children with a stimulating home environment [8–10]. Further exacerbating this issue could be the possibility that these families have worse sociocultural traits [4, 9, 11, 12]. A heterogeneous sample of patients with severe developmental disabilities or children getting special education has frequently been included in studies throughout the literature that demonstrate children with developmental delay have a poorer home environment than typically developing children [4, 9, 10, 13–15]. Initiating special education for a child can provide resources and training to help families understand their child’s unique needs, support their development, and implement strategies that promote learning [16, 17].

The aim of our study was to compare the home environment, screen time, and sociodemographic characteristics of preschool children monitored in a developmental pediatrics clinic for developmental delays who were not receiving special education support with those of typically developing children.

## 2. Materials and methods

### 2.1. Participants

The research was carried out at Hacettepe University Division of Developmental Pediatrics and the general pediatric outpatient clinic of Ankara 29 Mayıs State Hospital. It had been authorized by the Hacettepe University Faculty of Medicine Ethics Committee (GO 22/1000). The study comprised of 50 children aged 3–6 years who were admitted to Hacettepe University İhsan Doğramacı Children’s Hospital Developmental Pediatrics outpatient clinic between 01.01.2023 and 31.06.2023 due to developmental risks or concerns and who had a delay in at least one domain of development. The patients had no known genetic, neurodevelopmental or neurometabolic diseases. None of them had received individualized or structured special education support from special education and rehabilitation centers in any developmental domain (cognitive, language-relationship, motor, or socio-emotional) prior to this study. The control group included 70 children with typical development who attended Ankara 29 Mayıs State Hospital general pediatrics clinic. The Ages and Stages Questionnaire (ASQ) was utilized by physicians and experienced child development professionals to conduct developmental assessments on every child. The parents completed the Home Environment Questionnaire, and the sociodemographic details of the families were recorded. The Hollingshead-Redlich Scale was employed to ascertain the families’ socioeconomic-sociocultural levels. The scale is based on the educational and occupational status of the parents [18].

### 2.2. Evaluation tools

#### 2.2.1. The Ages and Stages Questionnaire (ASQ)

The ASQ, one of the most commonly used developmental screening tools in pediatrics, evaluates communication, gross motor, fine motor, problem solving, and personal-social development in children aged 3 to 72 months [19]. The ASQ was administered via parent interviews in conjunction with the literature in this study, although it was designed to be completed by parents [20]. A child whose score is within the shaded part of the scoring bar graph (−2 SDs below the mean) is regarded as having failed the domain. The current study used the Ages and Stages Questionnaire for Turkish Children (ASQ-TR) to evaluate the children’s development. The sensitivity and specificity of ASQ-TR are 0.94 and 0.85, respectively. The use of two or more abnormal ASQ domains as a positive screen in the adaptation research was suggested [21].

#### 2.2.2. The Home Environment Questionnaire (HEQ)

The HEQ was used to assess how developmentally stimulating the children’s homes were. The 17-item HEQ asks about children’s availability of toys, books, and CDs, as well as includes questions regarding activities like reading books to them, taking children to outside activities (parks, exhibitions, museums, etc.), and teaching them colors, shapes, numbers, and letters [22, 23]. The home observation for measuring the environment (HOME) scale was used to add 14 items to the scale, extending its content. Some of these items included the availability of jigsaw puzzles, toy blocks, crayons, and children’s songs or poems [24]. The number of stimulating materials in the home environment (e.g., the number of children’s books), activity frequency (e.g., how often do they read to their children, how often do they take them to places where they can play with other children? ), and whether educational activities are performed (e.g., does anyone else help your child learn numbers, colors, shapes, and letters) are all thoroughly investigated. The 31 items included in the extended scale were completed by the researchers by asking the parents directly. An HEQ score was derived based on the number of materials and activity frequencies reported by parents. The original coding scheme of the HEQ[26] [22] was adopted for this study, with all scores being “dummy scored” on a scale from 0 to 5 based on the available options. To illustrate, households with 10 or more children’s books were assigned a score of 3; 3 to 9 books were scored as 2; 1 or 2 books as 1; and no books as 0. These individual scores were then added together to generate a home stimulation score. The validity of this version of the scale has been evaluated in a comprehensive study. The scale’s convergent validity is supported by relationships with household chaos (r = −.38, p < .01), while criterion validity is supported by associations with parent education (r = .33, p < .01) and family income (r = .40, p < .01) [25]. Additionally, responses to the item in the home environment questionnaire—’Children act poorly sometimes and well other times. Have you ever struck your child because they irritated you in the preceding week? If so, how often did it occur on average?’—were analyzed in greater detail. A ‘yes’ response to this question was considered indicative of the presence of physical abuse. Children’s average daily screen time and background television exposure were obtained through detailed questions posed to parents.

### 2.3. Statistical analysis

IBM SPSS Version 22.0 was used to perform the statistical analysis. The numerical variables were summarized as mean, standard deviation (SD), and/or median [min–max], while categorical variables were given as frequencies and percentages. Differences between groups in continuous variables were determined utilizing an independent sample t-test or Mann-Whitney U test, as appropriate. The Pearson chi-square test was used to determine these differences for categorical variables. The relationship among continuous variables was determined by the bivariate Pearson and Spearman correlation coefficients. A multiple linear regression model was performed to test whether family sociodemographic characteristics and child developmental traits predict the home stimulation score. Variables that had a statistically significant effect on the home stimulation scores (p < 0.05) were included in this model. Assuming that the presence of developmental delay has a moderate effect (0.60) on home environment stimulation, a sample size of 120 was calculated, with 95% power and a 5% type 1 error level. A p-value of less than 0.05 was considered significant.

## 3. Results

The mean age of 50 children with developmental delay was 48.94 (±9.87) months, and there was a significant difference with the 56.88 (±9.06) months mean age of the 70 typically developed children included in the study (p < 0.001). All of the participants were born at term, and there were no differences in gender, gestational age, or birth weight between the two groups. Compared to children with typical development, the educational attainment of both parents and the socioeconomic level of their households were significantly lower for those with developmental delay. Table 1 presents the sample’s sociodemographic characteristics. The analysis of developmental outcomes revealed that among the 50 children with developmental delays, delays were most frequently observed in the communication domain (45 children, 90%), followed by the problem-solving domain (39 children, 78%), fine motor domain (33 children, 66%), personal-social domain (25 children, 50%), and gross motor domain (6 children, 12%). Among the children, 6 had delays in a single area, 14 in two areas, 13 in three areas, 11 in four areas, and 6 exhibited delays across all developmental areas.

In the group of children with developmental delay, the average home stimulation score was 30.3 ± 9.16, whereas it was 44.7 ± 4.18 for typically developing children (p < 0.001). The groups’ average daily screen time was 2.1 ± 1.4 h and 4.1 ± 2.5 h, respectively, and it was significantly higher for the children with developmental delay (p < 0.001). A negative correlation was observed between daily average screen time and home stimulation scores in both children with typical development and those with developmental delay (p = 0.042, p = 0.034, respectively). A significant finding of the study revealed that 34% of parents of children with developmental delay reported physically abusing their children (Table 2).

The associations between the families’ sociodemographic characteristics and home stimulation scores were evaluated. Significantly higher home stimulation scores are observed in both typically developing children and children with developmental delay whose mothers have attained an educational level beyond high school (respectively, p = 0.029, p = 0.006). Regardless of the group, kindergarten attendance was associated with higher home stimulation scores than non-attendance (p = 0.045, p < 0.001) (Table 3). The number of members in the family and the number of siblings did not significantly affect the home stimulation score of typically developing children (p = 0.384 and 0.657, respectively), however, there was a positive link with the family’s socioeconomic status (p = 0.010). Additionally, it was found that the number of siblings and family members had a negative correlation with the home stimulation score (p = 0.001, p < 0.001) and a positive correlation with the family’s socioeconomic status for children with developmental delay (p < 0.001).

The home stimulation scores of children with developmental delay decreased as the number of failed development domains in the ASQ increased (p < 0.001, correlation coefficient −0.538). Stepwise multiple regression analyses were used to identify the significant risk factors related to home stimulation scores, so variables such as maternal and paternal education levels, the number of siblings and household members, the family socioeconomic status, and the presence of developmental delay were included in the model. In the final model, the presence of developmental delay (p < 0.001, CI 12.629–7.927), the family socioeconomic status (p = 0.002, CI 3.813–1.486), and the number of members in the family (p = 0.001, Cl 2.595–0.708) were significantly associated with home stimulation scores (Table 4).

## 4. Discussion

This study revealed that home stimulation scores for children with developmental delay were significantly lower compared to those for their typically developing peers. In Van der Schuit’s et al. study, the quality of the home literacy environment was notably inferior for preschool children with intellectual disabilities, primarily those with cerebral palsy, compared to that of children without such disabilities [10]. Disability can contribute to stress and strained parent-child relationships within families, as shown by a few studies investigating the home environment of children with impairments such as orthopedic and visual. The caliber of the home environment provided can also vary depending on the level of disability [8, 26, 27]. Conversely, some studies indicate that there is no significant difference in the home environments of children receiving special education and those who are typically developing [13, 14]. In a comprehensive study conducted by Sucuoğlu et al., it was found that the home environments of children with disabilities were less favorable compared to those of their healthy peers. Additionally, the study highlighted the positive impact of inclusive preschools on both families and the quality of the home environment [9]. In this context, our research findings align with existing literature and are significant since they encompass a patient group that has not received special education support in any developmental domain.

Children with developmental delay are substantially more likely than their peers to face established social determinants that lead to worse health and developmental outcomes, such as low socioeconomic status and poverty [11, 12]. Socioeconomic status, encompassing factors such as income, wealth, and education, is crucial for promoting children’s health and development. It impacts familial aspects like adequate nutrition, quality caregiving, effective parenting practices, and the provision of a stimulating home environment [11, 25, 28]. In our study, in line with the literature, the family’s socioeconomic status was a key determinant of the home environment [4, 7, 11, 13]. Furthermore, children of highly educated mothers often benefit from increased exposure to stimulating learning opportunities and enhanced sensitivity and nurturing care from their mothers [29]. A positive correlation between maternal education level and the home stimulating score was observed in both groups in our research. The absence of a negative impact on the home stimulation score from an increased number of household members and siblings in typically developing children, compared to those with developmental delay, may be explained by higher socioeconomic status and maternal education levels. Families could be encouraged to optimize their home environment to facilitate the child’s exploration and development, create and utilize low-cost toys, and increase their time spent with the child, in conjunction with referring children with developmental delays to early intervention services [4, 9].

The World Health Organization and the American Academy of Pediatrics advise against any screen time for infants and toddlers up to age 2 years and recommend limiting screen time to no more than one hour per day for children aged 2 to 5 years [30, 31]. In our study, screen time exceeded the recommended durations for both typically developing children (2.1 ± 1.4 h) and children with developmental delay (4.1 ± 2.5 h). Although research on screen use in children is quite heterogeneous due to methodological and cultural differences, our findings generally support the existing literature [32–35]. Among the children in this study, 50% of those with typical development and 12% of those with developmental delay complied with screen use recommendations. Many studies observe that preschool children often exceed the recommended screen time limits, even though these proportions vary across societies based on sociocultural characteristics [36, 37]. Our study also underscores a significant negative association between screen time and home stimulation scores. Furthermore, given the evidence from several studies showing the harmful effects of media on young children, such as violence, delayed language development, cognitive impairments, attention issues, obesity, and sleep problems, it is essential to inform parents about these risks [31, 34, 38].

Adverse childhood experiences (ACEs), particularly during the crucial phase of significant brain plasticity and development in early childhood, have long-lasting detrimental impacts on physical and mental health, as well as overall developmental trajectories throughout life [39, 40]. Children with chronic physical health problems [41], special educational needs [39, 42], and more severe disabilities [43, 44] are at an increased risk of child abuse, neglect, maltreatment, and exposure to ACEs compared to their healthy peers. The global prevalence of children experiencing physical domestic and familial violence as victims was 17.3%, with the highest prevalence rates observed in West Asia and Africa at 42.8% [45]. Child abuse and neglect occur in 3% to 10% of individuals with disabilities in the United States [44]. Due to differences in the level of societal development, various studies report prevalence rates ranging from 0.09% to 71% [41, 43]. A notable finding in our study is that 34% of children with developmental delays, who do not have severe disabilities, have been subjected to physical violence. Empirical epidemiological research on child abuse and physical violence in Turkiye is limited, with most cross-sectional studies focusing on school-aged children and adolescents [46, 47]. A study on 6–12-year-olds with Specific Learning Disorders (SLDs) found that 51.9% experienced physical violence, significantly more than their typically developing peers [48]. There is a pressing need for research encompassing early childhood and children with developmental delays. A range of individual, interpersonal, community, and societal factors has been implicated in the risk of child abuse and physical violence. Several risk factors have been identified, including having special health care needs or disabilities, exposure to multiple adult caregivers, poverty, low parental education levels, poor parental mental health and substance use, intimate partner violence within the home, neighborhood crime and violence, concentrated disadvantage, and broader economic policies and trends. Conversely, certain individual characteristics, such as self-regulation skills and social competence, as well as positive parental mental health, social support, the availability of neighborhood services for parents and families, and supportive social and economic policies, have been shown to function as protective factors [39] Children with chronic illnesses or disabilities may place greater emotional, physical, economic, and social demands on their families. Parental stress may be heightened, increasing the risk of poor mental health outcomes. In children with developmental delays, cognitive or communication difficulties, co-occurring behavioral problems, and unmet parental expectations may contribute to frustration, leading some caregivers to resort to inappropriate physical punishment [44]. In this context, home visitation programs targeting high-risk populations, such as children with developmental delays, along with the development of social, economic, and educational policies addressing risk and protective factors at the community level, are recommended [39, 44, 49]. Additionally, it is crucial for all healthcare professionals, particularly pediatricians, to recognize the signs and symptoms of child abuse, screen for it during each health visit, remain vigilant, make accurate diagnoses, and ensure appropriate reporting [42, 44].

Addressing the home environment and screen time at the initial admission of children with developmental delay and those at risk who have never received special education is a notable strength of this study. Given the rising incidence of visits due to developmental issues in pediatric and pediatric subspecialty outpatient clinics, it is crucial to inquire about the home environment of these children and implement early interventions. The limitations of our study include its small sample size and cross-sectional design.

Children presenting with developmental delay represent a patient group that is commonly encountered in clinical practice by both primary care providers and pediatric and subspecialty healthcare professionals. Both typically developing children and those referred for special education require an evaluation and improvement of their home environments. Guiding families with lower income and education levels by providing information and recommendations on child development, as well as directing them to family intervention services, can help mitigate persistent educational inequalities among vulnerable families. Furthermore, the high levels of physical violence exposure among children with developmental delay who do not have severe delays or chronic conditions highlight a significant concern, emphasizing the need for families to be thoroughly questioned about this issue.

## Figures and Tables

**Table 1:** Sociodemographic characteristics of the children with typical development and developmental delay.

Variable, n (%), mean (± SD) or median (min–max)	Typically developing children (n = 70)	Children with developmental delay (n = 50)	p value

**Age (months)**	56.88 (±9.06)	48.94 (±9.87)	**p < 0.001**

**Sex (male)**	39 (55.7)	37 (74)	0.063

**Gestational age**	39 (37–41)	39 (38–40)	0.932

**Birth weight (grams)**	3350.58 (±540.70)	3241.90 (±474.13)	0.249

**No chronic disease**	69 (98.5)	45 (90)	**0.034**

**Maternal age**	35.1 (±5.38)	33.3 (±5.88)	0.087

**Maternal education level**			
**Illiterate/unschooled**	0 (0)	5 (10)	
**Primary school**	3 (4.3)	21 (42)	
**High school**	22 (31.4)	14 (28)	**p < 0.001**
**University**	45 (64.3)	10 (20)	

**Employed mothers**	39 (55.7)	8 (16)	**0.001**

**No maternal chronic disease**	69 (98.5)	40 (80)	**0.001**

**Paternal age**	38.1 (±5.68)	37.9 (±7.94)	0.624

**Paternal education level**			
**Illiterate/unschooled**	0 (0)	2 (4)	
**Primary school**	2 (2.8)	18 (36)	
**High school**	20 (28.6)	19 (38)	**p < 0.001**
**University**	48 (68.6)	11 (22)	

**Employed Fathers**	67 (95.8)	46 (92)	0.204

**No paternal chronic disease**	68 (97.1)	46 (92)	0.204

**Number of members in the family**	4 (3–6)	4 (3–10)	**p < 0.001**

**Number of siblings**	1 (0–2)	1 (0–4)	**0.002**

**Hollingshead-Redlich Scale**			
**I**	2 (2.8)	0 (0)	
**II**	34 (48.6)	6 (12)	
**III**	19 (27.1)	14 (28)	
**IV**	13 (18.6)	18 (36)	**p < 0.001**
**V**	1 (1.4)	12 (24)	
**VI**	1 (1.4)	0 (0)	

**Table 2:** Home environment characteristics of the children with typical development and developmental delay.

Variable, n (%), mean (± SD) or median (min–max)	Typically developing children (n = 70)	Children with developmental delay (n = 50)	p value
**Weekday screen time (hours)**	1.92 (±1.47)	4.08 (±2.62)	**p < 0.001**
**Weekend screen time (hours)**	2.30 (±1.70)	4.14 (±2.82)	**p < 0.001**
**Meeting screen time guidelines**	35 (70)	6 (12)	**p < 0.001**
**Background television exposure (hour)**	3.88 (±2.61)	9.68 (±5.5)	**p < 0.001**
**TV on all day**	1 (1.42)	24 (48)	**p < 0.001**
**Attending kindergarten**	48 (68,6)	14 (28)	**p < 0.001**
HEQ* **score**	44.7 (±4.18)	30.3 (±9.16)	**p < 0.001**
**Physical abuse (yes)**	1 (1.42)	17 (34)	**p < 0.001**

* Home environment questionnaire

**Table 3:** The association between the HEQ scores and sociodemographic characteristics.

Variable, n (%), mean (± SD) or median (min–max)	HEQ scores for typically developing children (n = 70)	p value	HEQ scores for children with developmental delay (n = 50)	p value

**Maternal education level**				
**< High school**	43.28 (±4.55)	**0.029**	28.5 (±8.98)	**0.006**
**>High school**	45.4 (±3.78)		37.2 (±6.42)	

**Mother’s working status**				
**Employed**	43.9 (±4.20)	0.167	36.1 (±7.05)	**0.039**
**Unemployed**	45.3 (±4.10)		29.1 (±9.16)	

**Paternal education level**				
**< High school**	43.27 (±4.14)	0.066	28.2 (±8.67)	**0.030**
**>High school**	45.30 (±4.07)		37.5 (±7.17)	

**Attending kindergarten**				
**Yes**	45.35 (±4.20)	**0.045**	38.3 (±7.55)	**p < 0.001**
**No**	43.27 (±3.83)		27.1 (±7.76)	

* Home environment questionnaire

**Table 4:** Multiregression analysis of the factors effecting home stimulation score.

Variables	Unstandardized Coefficients Beta	p value	95% CI
**Presence of developmental delay**	−10.278	< 0.001	12.629–7.927
**Hollingshead Redlich scale**	−2.650	< 0.001	3.813–1.486
**Number of members in the family**	−1.652	0.001	2.595–0.708

* CI confidence intervals
